# Potent synthetic and endogenous ligands for the adopted orphan nuclear receptor Nurr1

**DOI:** 10.1038/s12276-021-00555-5

**Published:** 2021-01-21

**Authors:** Yongwoo Jang, Woori Kim, Pierre Leblanc, Chun-Hyung Kim, Kwang-Soo Kim

**Affiliations:** 1 Department of Biomedical Engineering, Hanyang University, Seoul, 04736 Korea; 2 Department of Psychiatry, McLean Hospital, Harvard Medical School, Belmont, MA 02478 USA

**Keywords:** Drug discovery, Transcriptional regulatory elements

## Abstract

Until recently, Nurr1 (NR4A2) was known as an orphan nuclear receptor without a canonical ligand-binding domain, featuring instead a narrow and tight cavity for small molecular ligands to bind. In-depth characterization of its ligand-binding pocket revealed that it is highly dynamic, with its structural conformation changing more than twice on the microsecond-to-millisecond timescale. This observation suggests the possibility that certain ligands are able to squeeze into this narrow space, inducing a conformational change to create an accessible cavity. The cocrystallographic structure of Nurr1 bound to endogenous ligands such as prostaglandin E1/A1 and 5,6-dihydroxyindole contributed to clarifying the crucial roles of Nurr1 and opening new avenues for therapeutic interventions for neurodegenerative and/or inflammatory diseases related to Nurr1. This review introduces novel endogenous and synthetic Nurr1 agonists and discusses their potential effects in Nurr1-related diseases.

## Neuropharmacology: a potential treatment strategy for dopamine-related brain disorders

Compounds that activate a gene-regulating protein called Nurr1 could help treat Parkinson’s disease and other brain disorders related to abnormal dopamine signaling. A team led by Kwang-Soo Kim from Harvard Medical School, Belmont, USA, reviews the various compounds, both synthetic and natural, known to enhance the activity of Nurr1. This protein binds DNA to regulate the expression of target genes and was long thought not to have such triggering molecules or ligands, which have recently been identified by the team. These activating compounds seem to protect dopamine-producing neurons from cell injury in various Parkinson’s-related cell and animal models. Given Nurr1’s role in the development, differentiation, and survival of dopaminergic neurons in the midbrain, these compounds could help boost the capacity of malfunctioning dopaminergic neurons in patients with Parkinson’s disease, schizophrenia, bipolar disorder, and other neuropsychiatric conditions.

## Introduction

In general, a nuclear receptor is a ligand-regulated transcription factor that regulates downstream gene transcription in the nucleus in response to binding to its specific ligand(s)^1^. Nuclear receptors with no known specific ligand(s) are referred to as orphan nuclear receptors^2^. Until recently, the transcription factor Nurr1, which belongs to the NR4A subfamily, was considered an orphan nuclear receptor. The NR4A subfamily of nuclear receptors includes Nur77 (NR4A1), Nurr1 (NR4A2), and Nor1 (NR4A3)^3,4^. All family members have a ligand-binding domain (LBD) that shares sequence homology with the LBDs of canonical nuclear receptors^5,6^. Nevertheless, it was widely believed that Nurr1 is constitutively active in a ligand-independent manner^5,6^. In fact, crystal structural analysis revealed that the Nurr1-LBD lacks a classical binding pocket for coactivators and/or ligands due to the tight packing of bulky hydrophobic side chain residues^7^. However, recent NMR solution and HDX-MS characterizations revealed that the Nurr1-LBD is highly dynamic with high solvent accessibility, showing a change in structural conformation more than twice on the microsecond-to-millisecond timescale^8^. Numerous researchers are gradually identifying and validating the endogenous and synthetic ligands that activate Nurr1. Furthermore, recent characterization of Nurr1’s endogenous ligands has aroused further interest in the development of Nurr1 agonists and therapeutic drugs for diseases related to the receptor^9,10^. Therefore, this review focuses on putative ligands known to induce/enhance Nurr1 transcriptional activity.

In the midbrain, Nurr1 is indispensable for the differentiation, maturation, and maintenance of midbrain dopaminergic neuron clusters (referred to as A9 and A10), which reside in the ventral tegmental area (VTA) and the substantia nigra pars compacta (SNpc), respectively^11,12^. Nurr1 is known to regulate the transcription of dopamine-related crucial genes, including tyrosine hydroxylase (TH), dopamine transporter (DAT), vesicular monoamine transporter 2 (VMAT2), and aromatic amino acid decarboxylase (AADC), which significantly influence striatal dopamine levels^5,13^. To modulate the expression of these genes, Nurr1 binds to the nerve growth factor-induced clone B (NGFI-B) response element specific sequence (NBRE; 5′-AAAGGTCA-3′) as a monomer or to the inverted repeat octanucleotide Nur response element (NurRE; 5′-TGACCTTT-n6-AAAGGTCA-3′) as a homodimer^14,15^. In addition, Nurr1 can interact with retinoid-X receptor alpha (RXRα), forming a heterodimer that binds to the DR5-specific sequence element (5’-GGTTCACCGAAAGGTCA-3’)^14,15^. Indeed, Nurr1-deficient mice fail to generate midbrain dopaminergic neurons^16^, and tamoxifen-induced disruption of Nurr1 in the midbrain of adult mice also results in a progressive reduction in the number of striatal dopaminergic neurons^17^. Thus, these findings support Nurr1’s critical roles in both the development and maintenance of midbrain dopaminergic neurons.

In addition, Nurr1 is also known to exert neuroprotective effects against neuroinflammation of dopaminergic neurons. The promoter region of Nurr1 contains the transcriptional binding site of cAMP-responsive element-binding protein (CREB), and its interaction induces the upregulation of Nurr1 transcripts. This CREB-mediated Nurr1 upregulation seems to be involved in neuroprotection as a regulatory mechanism downstream of CREB^18^. Thus, it is possible that G-protein coupled receptors that increase intracellular cAMP levels, such as brain-derived neurotrophic factor (BDNF) and prostanoid receptors, which are involved in the neuroprotective pathways for dopaminergic neurons, are highly associated with Nurr1. In neuroprotective pathways, Nurr1 is known to regulate the expression of various nuclear-encoded mitochondrial genes in dopaminergic neurons^17^. It was found that a significant reduction in the levels of superoxide dismutase 1 (SOD1), which is one of these mitochondrial genes, is strongly implicated in oxidative stress detoxification in Nurr1-ablated cells^17^. Furthermore, Nurr1 is known to transcriptionally repress proinflammatory genes in microglia and astrocytes upon exposure to bacterial lipopolysaccharide (LPS), suggesting that it exerts neuroprotection by suppressing neurotoxic proinflammatory genes in the brain during inflammation^19–21^. Taken together, this evidence indicates that Nurr1 transcriptionally promotes the expression of anti-inflammatory genes, represses the expression of proinflammatory genes, and protects dopaminergic neurons.

While Nurr1 is important for the maintenance and protection of dopaminergic neurons, it has been reported that Nurr1 expression in the brains of Parkinson’s disease (PD) patients and in the brains of rodent models of PD induced by forced expression of α-synuclein or exposure to neurotoxins (6-OHDA or MPTP) is downregulated^22–24^. To date, no mechanistic hypothesis has been put forth to explain this reduced expression. Therefore, the development of selective Nurr1 agonists offers the possibility to provide target-based therapeutic interventions for PD and other related diseases.

## Bicyclic compounds

Contrary to the existing concept that Nurr1 is constitutively active, several studies have attempted to identify potent Nurr1 agonists from established chemical libraries. Because Nurr1 binds to the promoter region of target genes, cell-based luciferase reporter assays using specific Nurr1-binding sequence(s) have been used for high-throughput screenings.

**Fig. 1:**
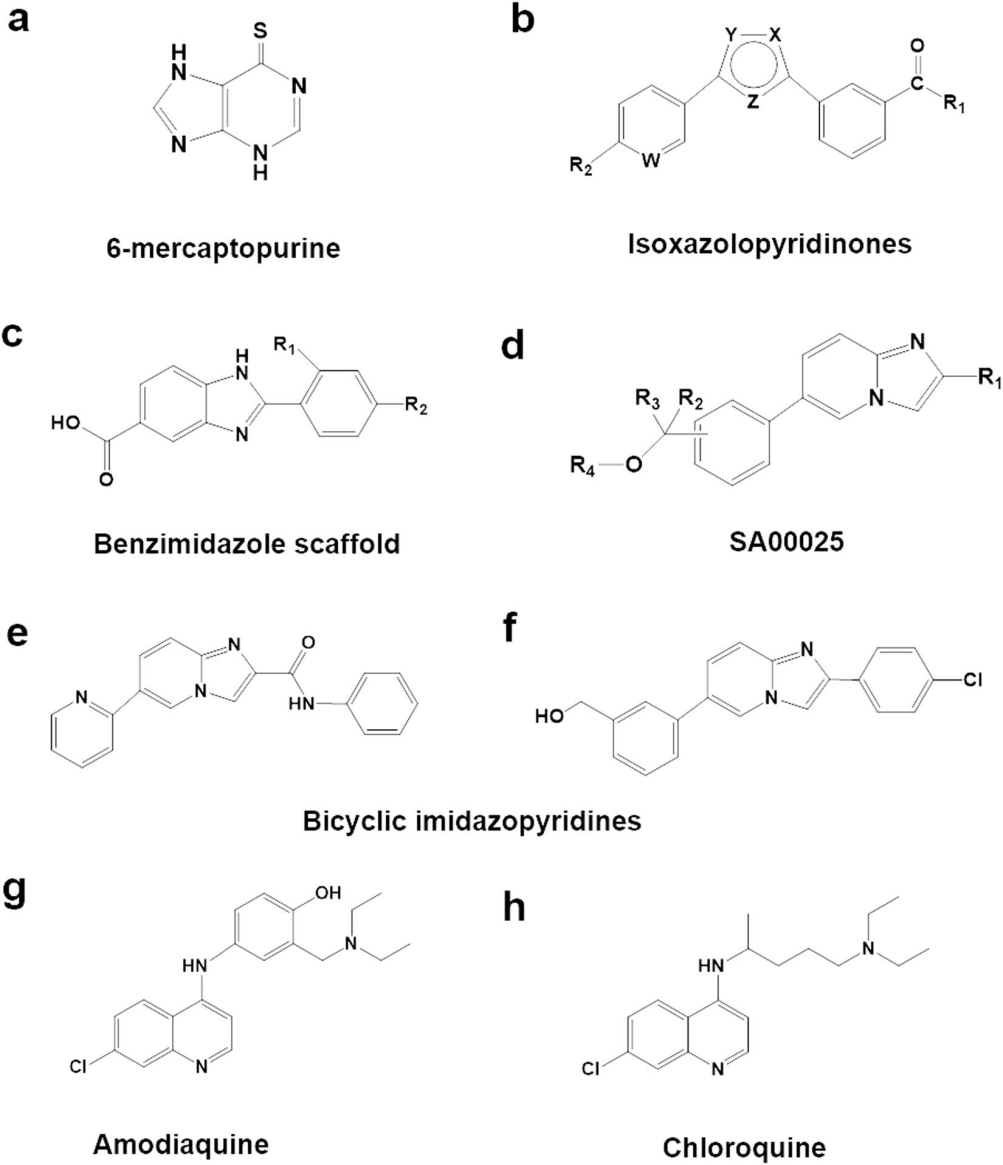
Chemical structures of synthetic activators of Nurr1. Synthetic activators for Nurr1 transactivation are 6-mercaptopurine (**a**), isoxazolopyridinones (**b**), benzimidazole scaffold (**c**), SA00025 (**d**), bicyclic imidazopyridines (**e,** **f**), amodiaquine (**g**), and chloroquine (**h**).

6-Mercaptopurine was the first Nurr1 agonist identified from a 340,800 compound prototypic library (Fig. 1a)^25^. 6-Mercaptopurine is commonly used for treating cancer and autoimmune diseases such as acute/chronic leukemia, ulcerative colitis, and Crohn’s disease^25–27^. It was shown to robustly enhance Nurr1 transcriptional activity using the NurRE target sequence in a concentration-dependent manner in CV-1 cells^25^. In the following year, reports indicated that bicyclic isoxazolopyridinone-based compounds increase Nurr1 reporter gene activity as a homodimer and a heterodimer using the NurRE and DR5 sequence elements, respectively (Fig. 1b)^28^. The molecular interaction between Nurr1-LBD and these compounds was demonstrated by a radiolabeled binding assay^28^. Additionally, these isoxazolopyridinones were shown to increase dopamine levels in the substantia nigra (SN) and striatumof OF1 mice at doses of 5 to 30 mg/kg^28^.

Based on the benzimidazole scaffold, a bicyclic compound of fused benzene and imidazole (Fig. 1c), Dubois and colleagues used combinatorial chemistry and structure-based drug design to develop a potential Nurr1 agonist^29^. They synthesized 3840 compounds harboring the benzimidazole scaffold and tested for potential agonists using a Nurr1 DNA binding domain (DBD)-containing luciferase construct in MN9D cells. To rule out the possibility of heterodimer activation of Nurr1 and RXRα, they also assessed luciferase activity in the RXRα-overexpressing cell line. Using this reporter assay, they identified three potent Nurr1 agonists active with an EC_50_ in the nanomolar range (8–70 nM)^29^. In 2008, the pharmaceutical company Sanofi Aventis introduced the bicyclic-based Nurr1 agonist SA00025, a 2-aryl-6-phenylimidazo[1,2-α]pyridine derivative (Fig. 1d)^30^. In vivo, oral administration of SA00025 (30 mg/kg) for 32 days protects dopaminergic neurons in a 6-OHDA-induced lesion rat model of PD exacerbated by inflammation^31^.

**Fig. 2:**
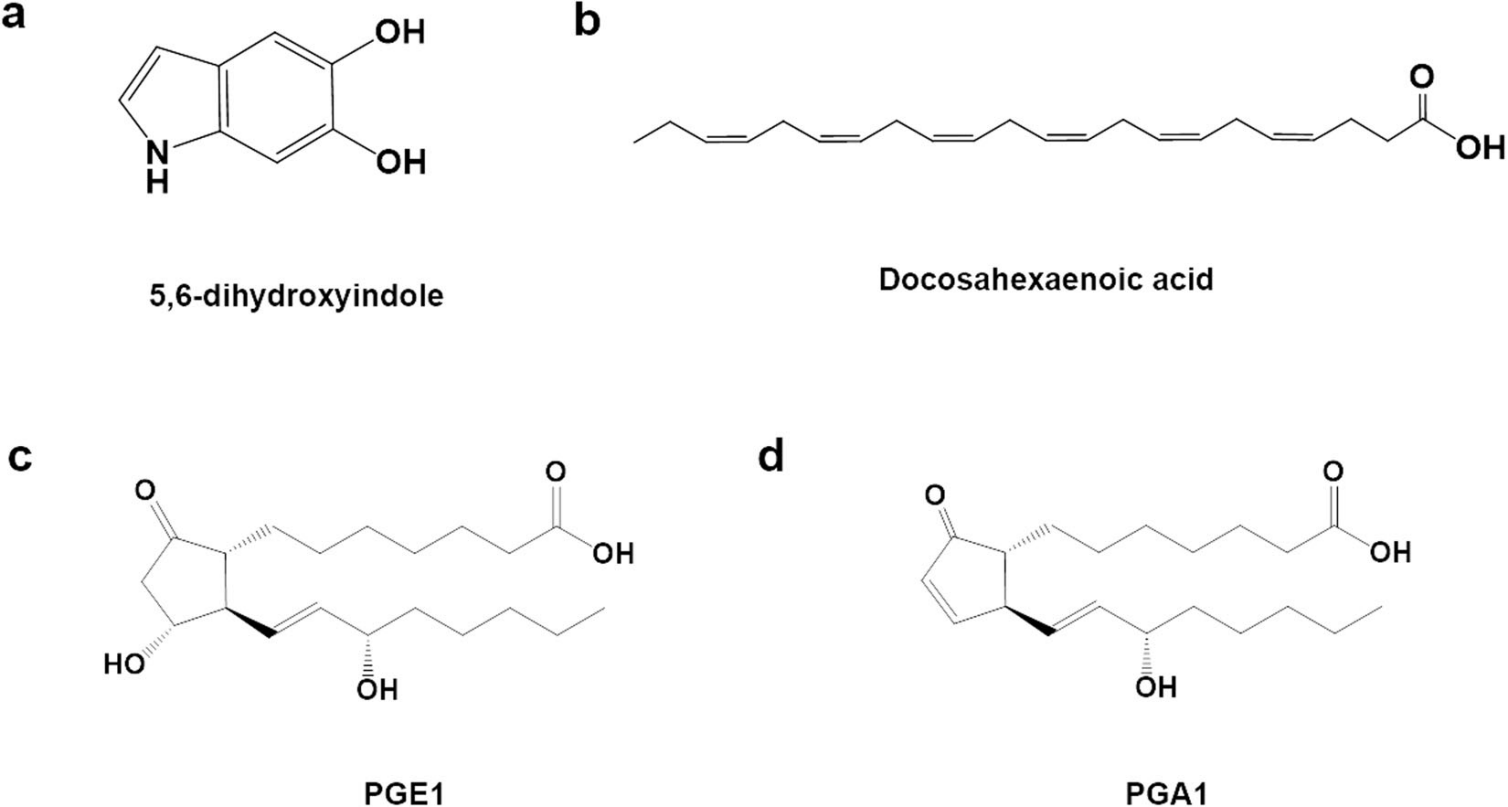
Chemical structures of endogenous ligands of Nurr1. Endogenous ligands for Nurr1 transactivation are 5,6-dihydroxyindole (**a**), docosahexaenoic acid (**b**), PGE1 (**c**), and PGA1 (**d**).

**Fig. 3:**
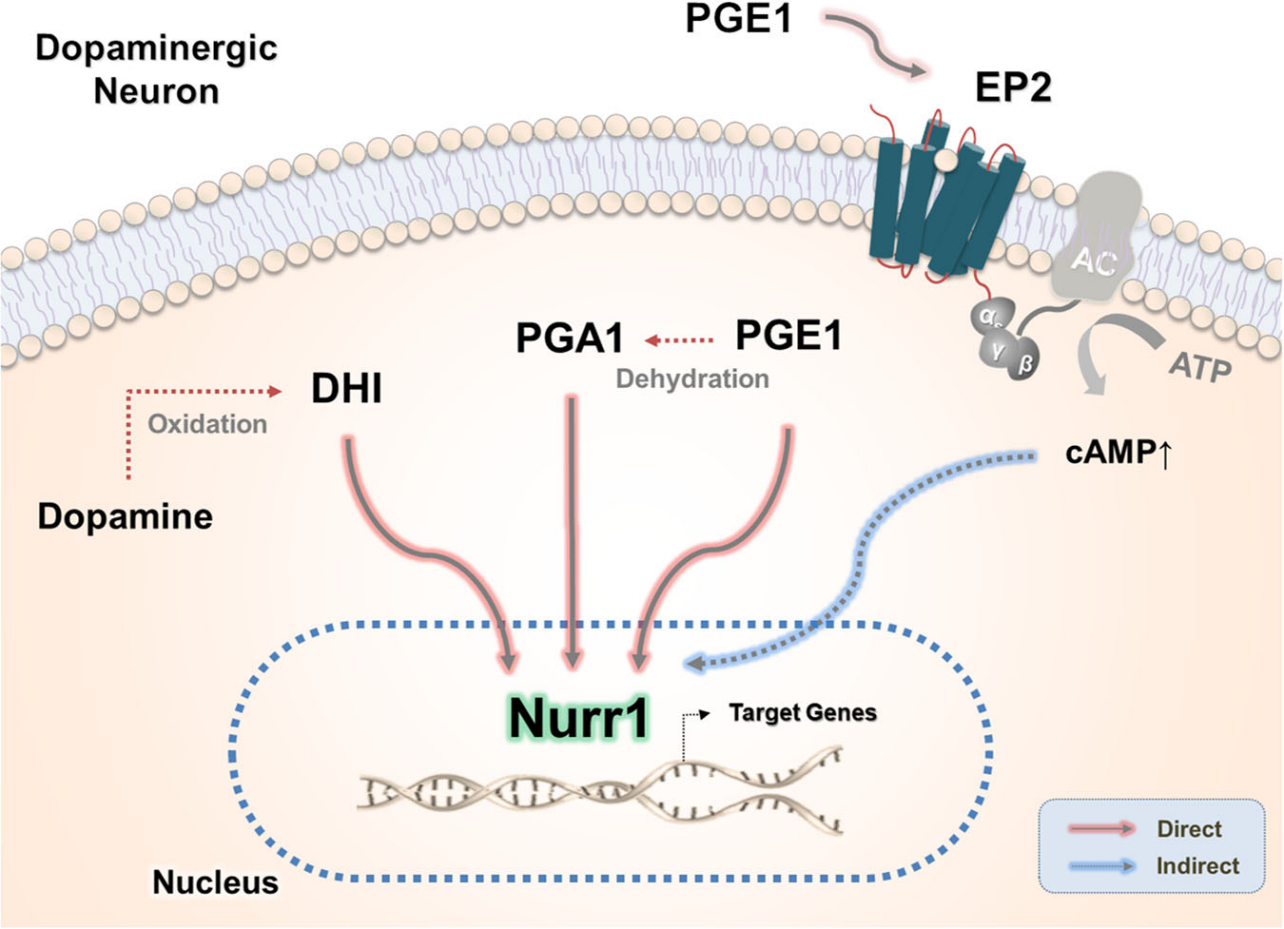
Endogenous ligands of Nurr1 in dopaminergic neurons.

**Fig. 4:**
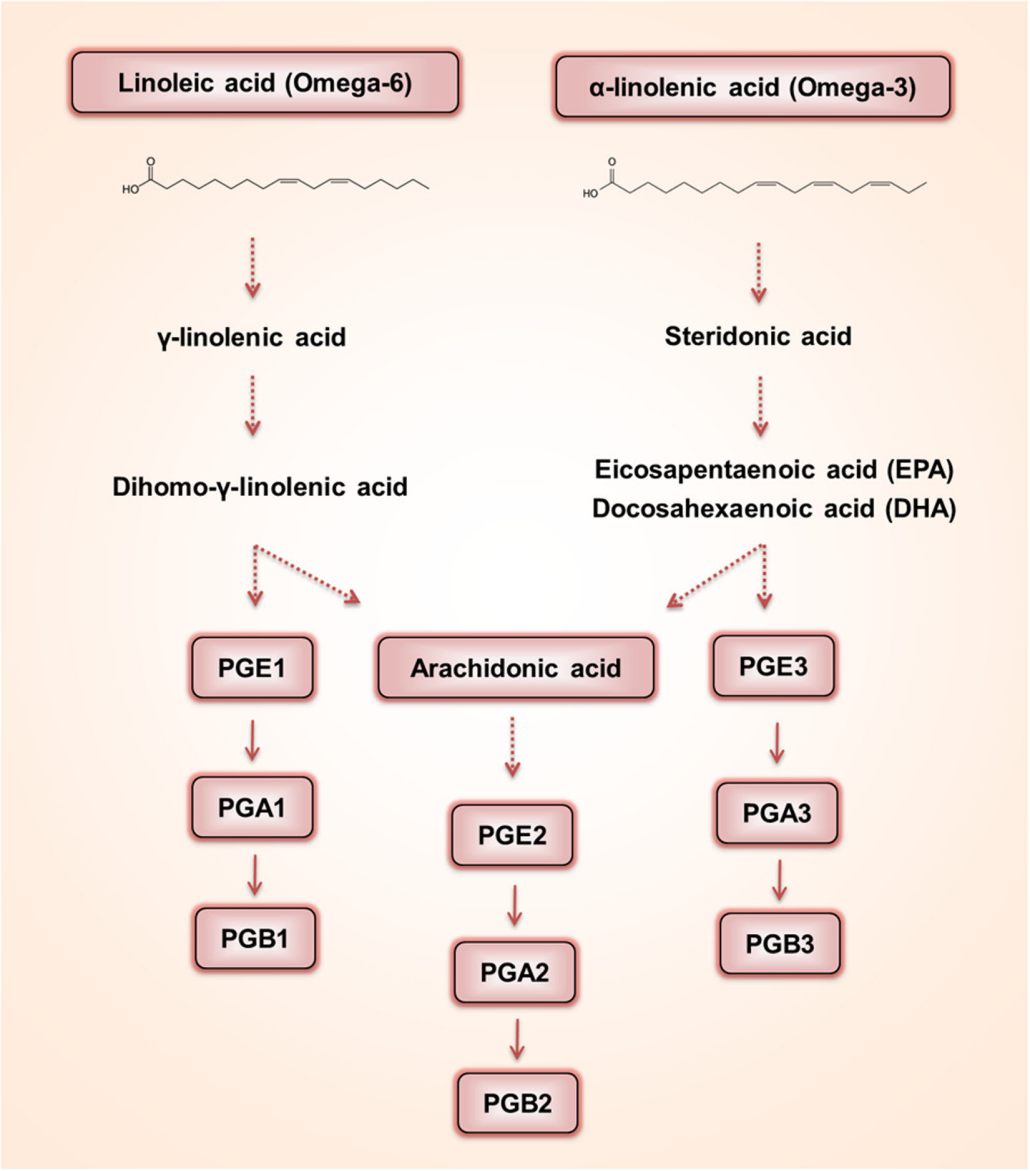
The pathway by which PGE1/2/3 and PGA1/2/3 are produced from omega 3 and 6 unsaturated fatty acids.

Recently, Lesuisse et al. found two additional bicyclic imidazopyridines that act as Nurr1 agonists through compound screening based on the NBRE sequence coupled to a luciferase reporter gene in the neuronal N2A cell line^32^. The identified imidazopyridine-derived compounds are N-phenyl-6-(pyrid-2-yl)imidazo[1,2-a]pyridine-2-carboxamide (Fig. 1e) and (3-[2-(4-chlorophenyl)imidazo [1,2-a]pyridin-6-yl]phenyl)methanol (Fig. 1f)^32^. They enhance Nurr1 transcriptional activity using the NBRE target sequence at an EC_50_ of 1 nM^32^.

## Amodiaquine (AQ) and chloroquine (CQ)

### Molecular interactions of AQ/CQ with Nurr1

Using a cell-based luciferase assay system, Kim and colleagues screened a chemical library composed of 960 FDA-approved drugs and identified 3 potential Nurr1 activators, namely, amodiaquine (AQ) (Fig. 1g), chloroquine (CQ) (Fig. 1h), and glafenine (Gla)^33^. Interestingly, all three compounds share an identical chemical scaffold, 4-amino-7-chloroquinoline, suggesting a specific structure-activity relationship for these Nurr1 activators. Using a luciferase-based reporter assay consisting of the DBD of the yeast transcription factor GAL4 fused to the Nurr1-LBD, AQ and CQ were shown to increase the transcriptional activities of Nurr1-LBD and full-length Nurr1 in the human neuroblastoma cell line SK-N-BE(2)C^33^. Moreover, using radiolabeled [^3^H]-CQ, the researchers showed saturable binding to Nurr1-LBD with a dissociation constant (*K*_d_) of 0.27 μM and a maximal binding capacity (*B*_max_) of 13.9 μM^33^. Unlabeled AQ competed for the binding of [^3^H]-CQ to Nurr1-LBD, indicating that AQ/CQ binds to similar Nurr1-LBD residues. Furthermore, nuclear magnetic resonance (NMR) spectroscopy also suggested the existence of molecular interactions between AQ/CQ and Nurr1-LBD. The comparison of Nurr1-LBD spectra for free and AQ-bound showed that multiple residues were shifted in the helix α2 region (His402, Ile403, Gln404, Gln405, Asp408, and Leu409) and the helix α11 region (Val468, Tyr575, and Asp580)^33^. CQ-bound spectra showed perturbed residues mostly in the helix α4 (Ser441) and the helix α12 region (Ile573, Ala586, Ile588, Lys590, Leu593, Asp594, Thr595, Leu596, and Phe598) of Nurr1-LBD^34^.

### Nurr1-dependent biological effect of AQ/CQ

Malaria is a serious tropical disease associated with high fever, chills, and flu-like symptoms and is typically transmitted by *Plasmodium* parasites through mosquito bites^35^. Originally, AQ/CQ was developed as anti-malaria drugs, and they are still commonly used to treat malaria^36^. Since AQ/CQ is known as active Nurr1 ligands, several studies have demonstrated the therapeutic effects of AQ/CQ based on Nurr1 modulation in several neurodegenerative and inflammatory diseases^33,34^.

The biological effects of AQ/CQ were demonstrated in the dopaminergic system. In cultured rat dopaminergic neurons, exposure to AQ was shown to significantly enhance the mRNA levels of Nurr1-target genes such as TH, DAT, VMAT, and AADC in a concentration-dependent manner^33^. Furthermore, this study revealed that CQ had neuroprotective effects against the neurotoxin 6-OHDA both in vitro and in vivo, resulting in significant improvements in PD-like motor behaviors in 6-OHDA-lesioned rats^33^. Hedya et al., using the rotenone rat model of PD, showed that the less toxic derivative hydroxychloroquine (HCQ) exerts an anti-inflammatory effect associated with increased Nurr1 expression, reduced activity of GSK-3β, and diminished expression levels of inflammatory mediators (NF-κB, TNF-α, and IL-1β)^37^. Consequently, treatment with HCQ resulted in amelioration of rotenone-induced impaired motor behaviors^37^.

Since Nurr1 is highly expressed in hippocampal neurons as well, Moon and colleagues attempted to clarify the function of Nurr1 and the effect of AQ in Alzheimer’s disease (AD), which is characterized by memory loss (dementia) caused by neuronal loss in the hippocampus starting in the early stage of disease^38,39^. By overexpressing and silencing *Nurr1* in the hippocampal area, the researchers demonstrated that Nurr1 ameliorates AD‐related pathological symptoms, including Aβ‐plaque deposition, in 5XFAD mice^38^. In line with these findings, 5XFAD mice treated with AQ exhibited marked improvements in typical AD pathogenesis, including deposition of Aβ plaques, neuronal loss, impaired adult hippocampal neurogenesis, and cognitive defects^38^. Further investigation of adult hippocampal neural stem cells determined that AQ increases hippocampal adult neurogenesis through cell cycle progression^40^.

Recently, Kinoshita et al. demonstrated AQ’s anti-inflammatory effect from a different perspective. Nurr1 is highly expressed in microglia/macrophages and astrocytes in the perihematomal area of the striata of mice with intracerebral hemorrhage (ICH)^41^. Intraperitoneal injection of AQ (40 mg/kg) after IHC induction prominently attenuated the activation of microglia/macrophages and astrocytes in the perihematomal area, consequently improving impaired motor deficits^41^. Given that CQ is commonly used to treat autoimmune diseases such as rheumatoid arthritis^42–44^, it is possible that Nurr1 is involved in the differentiation of naïve T cells into regulatory T cells, which are critical to immune homeostasis^45–47^. Indeed, Park et al. found that CQ facilitates differentiation into regulatory T cells via induction of the Foxp3 gene by Nurr1^34^. Intraperitoneal injection of CQ (50 mg/kg) was shown to mitigate colitis inflammation in a dextran sulfate sodium (DSS)-induced colitis mouse model, a valid animal model of inflammatory bowel disease^34^.

## 5,6-Dihydroxyindole (DHI)

### Molecular interactions of DHI with Nurr1

The biosynthesis and storage of the neurotransmitter dopamine are regulated by several enzymes and cofactors, all of which can control dopamine levels. In dopamine synthesis, TH is a rate-limiting enzyme that converts amino acid tyrosine to L-dopa^48,49^. Subsequently, L-dopa is converted to dopamine by the enzyme AADC^48,49^. The resulting dopamine is transported into the synaptic vesicle by VMAT2^50^. Released dopamine is recycled by DAT, which takes it back into the cytosol from the synaptic cleft^51^.

Nurr1’s involvement in dopamine homeostasis includes transcriptional regulation of key factors involved in dopamine synthesis (TH and AADC), vesicle packaging (VMAT2), and dopamine reuptake DAT, which could significantly influence striatal dopamine levels^5,13^. Therefore, it is possible that Nurr1’s activity in dopamine processing could be tightly regulated by dopamine, its precursors, and/or its metabolites to maintain dopamine homeostasis. In support of this possibility, using biophysical assays and X-ray crystallography, Bruning and colleagues found that the dopamine metabolite 5,6-dihydroxyindole (DHI) directly binds to Nurr1-LBD (Figs. 2a and 3)^9^. DHI forms a covalent adduct with Cys566 on helix H11 adjacent to the canonical ligand-binding pocket in well-characterized nuclear receptors^9^. The ligand electron density map and quantum mechanical calculations support the view that a chemical reaction between the Cys566 sulfur atom and the C2 atom of 5,6-indolequinone, the autooxidation product of DHI, leads to adduct formation^9^. These findings indicate that DHI binds as a quinone to the Cys566 residue of Nurr1. To investigate the effect of DHI on the transcriptional activity of Nurr1, a cell-based luciferase assay using a GAL4 DBD-fused Nurr1 construct was performed, and it was found that DHI induces the transcriptional activity of Nurr1-LBD in JEG3 cells, with 10 μM DHI having a significant effect and 100 μM DHI inducing a 1.6-fold increase in activation over basal levels^9^.

### Biological effects of the interaction of DHI with Nurr1

Little is known about the effect of the recently identified DHI on the dopaminergic system. Under oxidative stress, dopamine can be oxidized into two DHI and 5,6-indolequinone molecules, which are cytotoxic intermediates in the biosynthetic pathway of neuromelanin^52^. Thus, oxidative stress induces pathological accumulation of cytosolic dopamine in dopaminergic neurons. In particular, the dopamine oxidation product DHI could influence the deposition of α-synuclein aggregates in dopaminergic neurons, a pathogenic feature of PD^53^. Taken together, this evidence suggests that because excessive dopamine proportionally increases the levels of these reactive metabolites, it is likely that DHI-induced Nurr1 activation regulates the transcription levels of Nurr1 target genes, including those involved in the synthesis, packaging, and reuptake of dopamine.

To verify this possibility, the changes in the transcription levels of several Nurr1 target genes induced by DHI were evaluated in zebrafish. Exposure of zebrafish embryos (3 days post-fertilization) to 100 µM DHI resulted in a significant increase in VMAT2 and DAT transcript levels after 6 h as well as increases in VMAT2 and TH levels after 24 h^9^. However, the dependence of these changes on Nurr1 expression remains to be elucidated. Nurr1 knockdown and overexpression systems would offer conclusive evidence. In addition, the roles of DHI in the dopaminergic system should be further investigated in rodent models.

## Prostaglandin E1 (PGE1) and prostaglandin A1 (PGA1)

### Molecular interactions of PGE1 and PGA1 with Nurr1

Prostaglandins (PGs) are bioactive lipid metabolites derived from the metabolism of membrane polyunsaturated fatty acids^54^. Fatty acids are converted into well-known functional metabolites such as arachidonic acid, eicosapentaenoic acid (EPA), and docosahexaenoic acid (DHA)^55^. These unsaturated fatty acids have two long hydrocarbon tails, which may allow them to squeeze into the hydrophobic and narrow cavity of the canonical pocket of NR4A-LBDs. In fact, some remarkable studies have highlighted the interaction between unsaturated fatty acids and NR4A-LBDs. For example, a metabolomics-based study of immobilized Nur77-LBD revealed its interaction with unsaturated fatty acids, including arachidonic acid and DHA^56^. Using a pull-down assay, it was later shown that DHA also interacts with Nurr1-LBD (Fig. 2b)^57^. Titration of DHA with ^15^N-labeled Nurr1-LBD showed that DHA binding affects the methyl groups of Leu410, Ile483, and Ile486, which are imbedded in the Nurr1-LBD, resulting in a conformational change in the C-terminal helix 12^57^. In addition, DHA (12.5 and 50 μM) induced Nurr1 transcriptional activity using the NBRE target sequence in HEK293T and MN9D cells^57^. Further in-depth analysis revealed that unsaturated fatty acids, including arachidonic acid, linoleic acid, and oleic acid, bind to Nurr1-LBD and induce conformational expansion, which allows more ligands to access the ligand-binding pocket^8^.

The cyclopentenone PGA2 was the first PG identified as an active ligand of Nor1 and Nur77^58,59^. Kagaya and colleagues demonstrated a direct interaction between PGA2 and Nor1-LBD using a cell-free Biacore system. Moreover, they showed that PGA2 (10 μM) is a transcriptional activator of Nor1 in NIH3T3 cells^58^. In addition, incubation of recombinant Nur77 with PGA2-biotin allowed the identification of the PGA2-Nur77 complex through the covalent interaction between PGA2 and the Cys566 residue of Nur77^59^. In a Nur77-specific reporter assay, treatment with PGA2 caused dose-dependent transcriptional activation of Nur77 but not PGE2 in human bronchial epithelial (NHBE) cells^59^.

Most recently, an extensive study performed by Rajan et al. in which active compounds were isolated from homogenized mouse tissue extracts identified PGE1 (Fig. 2c) and PGA1 (Fig. 2d) as potent endogenous Nurr1 ligands (Fig. 3)^10^. Initially, using the DAD of the yeast transcription factor GAL4 fused to the Nurr1-LBD in a human neuroblastoma cell line (SK-N-BE(2)C), the authors found that treatment with several tissue extracts increased Nurr1 transcriptional activity. To isolate the active components from the brain extracts, they performed sequential purification using boiling, acetone precipitation, and ultrafiltration to identify the Nurr1 activity-enriched fractions. Finally, PGE1 and 8-iso PGE1 were identified from several candidate compounds in the final active fraction. In the process of crystallizing the Nurr1-LBD-PGE1 complex, the dehydrated PGE1 metabolite PGA1 was found to form a covalent adduct between the C11 atom of the cyclopentenone ring and the thiol group of the Cys566 residue of Nurr1-LBD. It is likely that crystallization conditions (MES buffer, pH 5.5) lead to the spontaneous dehydration of PGE1 to PGA1. This is supported by several reports showing PGE1 dehydration in acidic/basic environments^60,61^. An added intriguing feature of Nurr1-LBD binding to PGA1 is that the two long fatty acid tails of PGA1 are squeezed into a hydrophobic space surrounded by helices H4, H11, and H12, inducing the outward movement of helix H12 from the core of Nurr1 at an angle of 21°.

### Neuroprotective effects of PGE1 and PGA1 via interaction with Nurr1

As shown in Fig. 4, linoleic acid is a polyunsaturated omega-6 fatty acid with a final carbon-carbon double bond in the n-6 position, whereas α-linolenic acid is an omega-3 fatty acid with three double bonds. Both fatty acids are found in vegetable oils, oily fish, nuts, and seeds at different ratios^55^. PGE1 is synthesized from dihomo-γ-linolenic acid, which originates from linoleic acid (omega-6)^55^. PGE2 and PGE3 are enzymatically produced from arachidonic acids originating from linoleic acids and α-linolenic acid-mediated EPA/DHA. PGE1-3 is further converted to PGA1-3 by a nonenzymatic dehydration process^62,63^. In general, PGEs activate prostaglandin E receptors (EP1-EP4), which subsequently regulate the levels of intracellular second messengers, cAMP, and Ca^2+^, via G proteins^64–67^. Despite extensive investigations across various fields on the roles of PGs, little is known about the actions of PGE1 and PGA1 in dopaminergic neurons where Nurr1 is functionally expressed^16^.

Double immunohistochemistry for specific EP receptors and TH revealed that EP1/EP2 receptors and EP2/EP3 receptors are selectively expressed in dopaminergic and nondopaminergic neurons in the rat SN^68^. Unlike EP2- and EP3-selective agonists, a EP1 receptor-specific agonist (17-phenyl trinor PGE2) was shown to exert significant toxic effects on dopaminergic neurons at nanomolar concentrations^68^. Conversely, antagonism by EP1 receptor blockers (SC-19220 and SC-51089) was shown to protect dopaminergic neurons against 6-OHDA-induced toxicity in rat primary mesencephalic culture^68^. Moreover, 6-OHDA-induced dopaminergic neuronal death was alleviated by treatment with an EP2 receptor-selective agonist (butaprost)^69^. Taken together, these findings suggest that the EP2 receptor-mediated cAMP pathway seems to be crucially involved in the survival of dopaminergic neurons.

Based on reporter assays, both PGE1 and PGA1 induce transcriptional activation of Nurr1 in a dose-dependent manner. Interestingly, PGE1-induced Nurr1 activation is inhibited by an EP2-selective antagonist (PF-04418948), whereas PGA1-mediated activation is not. In fact, radiolabeled ligand binding assays with EP2 and Nurr1-LBD proteins using [^3^H]-PGE1 and [^3^H]-PGA1 demonstrated that PGE1 interacts with both EP2 and Nurr1 and that PGA1 binds only to Nurr1^10^. Taken together, this evidence indicates that PGE1 regulates the transcriptional activity of Nurr1 via two distinct mechanisms: an EP2-mediated pathway and direct binding to Nurr1-LBD. In contrast, PGA1 covalently binds to the Cys566 residue of Nurr1-LBD and activates its transactivation.

In the midbrain, the PGE1- and PGA1-mediated Nurr1 axis seems to be crucial for protection against dopaminergic neuronal degeneration. PGE1 (3 μM) or PGA1 (5 μM) treatment was shown to protect MN9D and N27-A cells from MPP^+^-induced toxicity in a Nurr1-dependent manner. PGE1 and PGA1 ameliorated both MPP^+^- and LPS-induced losses of TH-positive dopaminergic neurons in the nanomolar range in primary dopaminergic neuron-glia cocultures isolated from the rat embryonic ventral mesencephalic area. Moreover, intraperitoneal injection of PGE1 (2 mg/kg) or PGA1 (2 mg/kg) significantly rescued dopaminergic neurons in the SN and the striatum and alleviated impaired motor behaviors in a subchronic MPTP-induced animal model of PD.

## Conclusion and perspective

In this review, we introduced recent findings on potent synthetic and endogenous ligands of Nurr1 ligands, a nuclear receptor that has long been referred to as a ligand-independent nuclear receptor (Tables 1, 2). The actions of these activators and ligands strongly support the notion that Nurr1 is not an orphan nuclear receptor that is constitutively active in a ligand-independent manner. Recent in-depth characterization of the structural conformations of Nurr1-LBD and ligand-bound crystal structures further provides molecular and structural insights into the ligand-dependent roles of Nurr1 under physiological and pathological conditions. The dopamine oxidation metabolite DHI has been proposed as a regulatory factor in intracellular dopamine homeostasis through Nurr1 activation (Fig. 3). In addition, PGE1 and PGA1 have been proposed to mediate neuroprotection via activation of Nurr1 in neurodegenerative diseases such as PD and AD. It is likely that extracellular PGE1 regulates the transcriptional activity of Nurr1 via the EP2-mediated pathway, whereas intracellular PGE1/PGA1 bind directly to Nurr1-LBD, activating its transcriptional function (Fig. 4). While the EP2-mediated cAMP-dependent pathway may regulate Nurr1 function by upregulating its expression^70,71^, it is also possible that Nurr1’s protein stability and/or transcriptional activity may be regulated by altered levels of intracellular cAMP. Further studies are warranted for clarification of these mechanisms as well as for therapeutic development of these Nurr1 ligands.

**Table 1:** Identified Nurr1 ligands and their functionality.

Ligand	Ligand screening			Ligand functionality	Ref.
	Conc.	DBD	Cells	Functions and conc.	
6-Mercaptopurine	10, 50 μM	NuRE GAL4	HEK-293 cells	N.A.	^25^
Isoxazolopyridinones	1–1000 nM	NuRE DR5	Midbrain dopaminergic cell line	N.A.	^28^
Benzimidazole scaffolds	8–7 nM	NBRE	MN9D cells	N.A.	^29^
SA00025	0.01–1000 nM	NBRE	Neuro-2A cells	N.A.	^30^
Imidazopyridines	1 nM	NBRE GAL4	Neuro-2A cells	N.A.	^32^
AQ	30 μM	NL3 GAL4	SK-N-BE(2)C cells	• Exerts neuroprotective effects on primary DA neurons (5 μM) • Exerts neuroprotective effects in a 6-OHDA-lesioned rat model of PD (20 mg/kg) • Improves motor behavior deficits in a 6-OHDA–lesioned rat model of PD (20 mg/kg)	^33^
CQ	100 μM	NL3 GAL4	SK-N-BE(2)C cells	• Exerts neuroprotective effects on primary cultures of rat mesencephalic DA neurons (20 μM)	^33^
DHA	12.5, 50 μM	NBRE	HEK293T and MN9D cells	N.A.	^57^
DHI	10, 100 μM	GAL4	JEG3 cells	• Transcriptionally activates Nurr1 target genes in zebrafish (100 μM)	^9^
PGE1	0.001–10 μM	NL3 GAL4	SK-N-BE(2)C, MN9D, and N27-A cells	• Exerts neuroprotective effects on primary DA neurons (3 μM) • Exerts neuroprotective effects in a MPTP-lesioned mouse model of PD (2 mg/kg) • Improves motor behavior deficits in a MPTP-lesioned mouse model of PD (2 mg/kg)	^10^
PGA1	1–10 μM	NL3 GAL4	SK-N-BE(2)C, MN9D, and N27-A cells	• Exerts neuroprotective effects on primary DA neurons (5 μM) • Exerts neuroprotective effects in a MPTP-lesioned mouse model of PD (2 mg/kg) • Improves motor behavior deficits in a MPTP-lesioned mouse model of PD (2 mg/kg)	^10^

**Table 2:** Nurr1 ligand-mediated transcriptional changes.

Ligand	Target Genes	In Vitro/In Vivo Models	Ref.
Imidazopyridines	↑TH	Rat mesencephalic dopaminergic neurons	^32^
Imidazopyridines	↓IL-6	Poly(I:C)-lesioned mouse model of PD	^32^
AQ	↓IL-1β, ↓IL-6, ↓TNF-α, ↓iNOS	LPS-treated rat mesencephalic dopaminergic neurons	^33^
CQ	↑Nurr1, ↑Foxp3, ↑IL-2, ↑CD25, ↑FASL	Mouse primary naïve CD4^+^CD25^−^CD62L^high^ T cells	^34^
DHI	↑TH, ↑DAT, ↑VMAT2	Zebrafish	^9^
PGE1	↑TH, ↑DAT, ↑AADC, ↑VMAT2, ↑Pitx3, ↑c-Ret	MPP^+^-treated MN9D cells	^10^
PGE1	↑TH, ↑DAT, ↑AADC, ↑VMAT2	In vivo; midbrain	^10^
PGA1	↑TH, ↑DAT, ↑AADC, ↑VMAT2, ↑Pitx3, ↑c-Ret	MPP^+^-treated MN9D cells	^10^
PGA1	↑TH, ↑DAT, ↑AADC, ↑VMAT2	In vivo; midbrain	^10^

*TH* tyrosine hydroxylase, *IL-6* Interleukin-6, *IL-1β* Interleukin-1β, *TNF-α* tumor necrosis factor-α, *iNOS* INDUCIBLE nitric oxide synthase, *Nurr1* nuclear receptor related 1, *Foxp3* forkhead box P3, *IL-2* Interleukin-2, *CD25* Interleukin-2 receptor alpha, *VMAT2* vesicular monoamine transporter 2, *DAT* dopamine transporter, *AADC* aromatic l-amino acid decarboxylase, *VMAT2* vesicular monoamine transporter 2, *Pitx3* pituitary homeobox 3, *c-Ret* RET proto-oncogene

In conclusion, in vitro and in vivo studies have recently provided strong evidence that Nurr1-activating compounds/ligands have the potential to protect dopaminergic neurons in various PD-related cell and animal models. Consequently, modulators that enhance Nurr1 function have great potential to be developed as novel mechanism-based disease-modifying therapeutics for PD from a transcriptional regulation perspective.
